# Temperature-specific spectral shift of luminescing thermally altered human remains

**DOI:** 10.1007/s00414-023-03006-0

**Published:** 2023-05-13

**Authors:** Parnia Schariatmadary, Maurice C. G. Aalders, Roelof-Jan Oostra, Tristan Krap

**Affiliations:** 1 Amsterdam UMC location University of Amsterdam, Biomedical Engineering and Physics, Amsterdam, The Netherlands; 2 Amsterdam UMC location University of Amsterdam, Medical Biology, Section Clinical Anatomy and Embryology, Amsterdam, The Netherlands; 3 Faculty of Law and Criminology, Maastricht Institute for Criminal Studies (MICS), Maastricht University, Maastricht, The Netherlands

**Keywords:** Burned bones, Alternate light sources, Luminescence, Heat-induced changes, Forensic anthropology

## Abstract

Human bone has shown to have luminescent properties that remain throughout the phases of cremation, with the exception of fully carbonized bone, when excited with a narrow band light source. During this research, an alternate light source (420–470nm, peak at 445nm) was used to visualize and investigate latent details relevant for forensic investigations of human remains recovered at fire scenes. As fire is a destructive force, it induces a vast variety of physical and chemical alterations to all components of the bone, making the subsequent analysis and interpretation of burned human remains challenging. A spectral shift in emission bandwidth, from green to red, was previously observed when the exposure temperature increased from 700 to 800 °C. This spectral shift was reproduced on a total of 10 human forearms, divided into 20 segments, by burning at 700 °C and 900 °C in an ashing furnace. The shift of emission bandwidth caused only by an increase in temperature was furthermore investigated by colorimetric analysis, proving the spectral shift to be significant. By easily quantifying the spectral shift, substantiation is provided for the use of this technique in practice to improve the interpretation of heat induced changes of bone.

**Supplementary Information:**

The online version contains supplementary material available at 10.1007/s00414-023-03006-0.

## Introduction

There are many instances in which forensic investigations revolve around fire scenes. These include cases such as vehicle accidents, common house fires, but also mass disasters and homicides. In a homicide case, the perpetrator could use fire to destroy any potential evidence useful for biological or contextual reconstructions. Fire leads to a vast variety of changes to the body that make the subsequent recovery and analysis of the remaining material challenging. When a body is exposed to fire, the flames cause the muscles to contract, creating a characteristic pugilistic pose which exposes some anatomical areas more whilst shielding others [1]. Once the skin is destroyed, the fire reaches the subcutaneous fat tissue which is an excellent fuel when ignited [2]. It takes around 10 to 20 min to deflesh parts of the body [3]. Once the bones are exposed, heat-induced changes typically occur from the outer surface to the inside.

As fire has destructive powers it causes physical and chemical alterations to all components of the bone on a macroscopic (colour, weight loss, deformation) and molecular level (chemical composition, crystallinity) [4]. Two major physical changes occur when bone is exposed to fire; it first becomes charred and black and, later on, calcined and white. These changes are accompanied by molecular changes which are categorized into dehydration, decomposition, inversion and fusion [1], [2], [4-6]. The sequence of these stages always remains the same, but the rate and degree of the changes that occur with them depend on many factors, such as the temperature of the fire, exposure time, dioxygen availability, flesh coverage on the bones, positioning of the body and distance to the fire itself [2], [4], [7]. The four stages of molecular damage begin with dehydration. Previous literature states that dehydration occurs at temperatures between 100 and 600 °C [5]. However, the most current literature explains that water will evaporate around 100 °C, whereas the more structurally bound water will be removed at temperatures around 250 °C [8]. The bones keep their ivory/orange colour, similar to their unburned state. At 300–800 °C, decomposition sets in. The organic components are removed, and due to carbonization, the bone turns black. As a by-product of the decomposition, the water concentration increases again at temperatures of 400 °C, but evaporates immediately. During the last two stages above 700 °C, inversion and fusion, chemical and crystallinity changes of the inorganic bioapatite occur; the hydroxyapatite is altered, resulting in an increase of calcium oxide (CaO) and beta-tricalcium phosphate (β-TCP) [9], [10], and the apatite crystals grow until they fuse and coalesce together. The bone colour changes from grey to white once the carbon is burned out and the bone becomes calcined. It is important to note that these stages cannot be defined as steps that have a clear end and beginning, but that they overlap, creating a continuous and heterogenous process.

Heat-induced changes that are seen as a hindrance when reconstructing a crime scene can also hold evidentiary value; when patterns and alterations due to fire are fully understood, investigators can make substantiated interpretations and reconstructions of events at the fire scene, as well as a determination of a possible offence, with the goal of contributing information to cause and manner of death. For instance, colour is routinely used by forensic anthropologists as a tool to estimate the temperature of the exposed fire, as knowing the temperature can impact subsequent analysis steps like DNA and isotope analysis.

Current research is focusing on finding more methods for analysing burned human remains. It has been shown that both unburned and burned human bones exhibit luminescence when excited with a narrow bandwidth light source. The term photoluminescence is used to describe the observable emission of light of a substance when excited by light with a different wavelength and includes fluorescence and phosphorescence [11]. Light of a specific wavelength is absorbed, causing electrons to move into a higher energy state. As the electrons return to their initial ground state, they release the energy surplus in form of a photon [12]. Although fluorescence and phosphorescence differ in decay time they cannot easily be differentiated when using alternate (narrow bandwidth) light sources (ALS); thus, the term luminescence applies when using ALS.

The luminescence of biological traces has been a tool widely used in forensic investigations. The luminescent property of bone has been studied in the context of recovering human remains amidst fire debris [13], [14]. We have shown previously that the luminescence of burned bones undergoes a spectral shift from green to red once temperatures above 800 °C are reached [9]. A spectral shift can be defined as the shift in the luminescence emission spectrum of the bone caused by an increase in exposure temperature or exposure duration and its consequences on a molecular level, whilst excited under the same excitation spectrum. Using ALS, this difference in luminescence colour can be visualized and used by forensic anthropologists to aid in the estimation of exposure temperature once the bones become calcined and white [9]. However, colour can be observed differently between individuals, making it a subjective procedure, which is why a new approach that allows for a more objective interpretation is needed.

As this is a fairly novel topic of research, there is a lack in substantiation for the use of the luminescent property of skeletal human remains to infer more contextual information about the events of the fire scene, like exposure temperature and positioning. This overarching project aims to fill the knowledge gap on the luminescent properties of burned human bones with exploratory and fundamental experimental studies. The findings of this study will progress the understanding of the luminescence of thermally altered human bones and add a new objective method of using this property by tackling the question: how can the luminescent property of burned human bone and the implementation of ALS be used in an objective analysis method to aid in forensic investigations of fire scenes?

## Materials and methods

### Sample selection and native luminescence

**Fig. 1:**
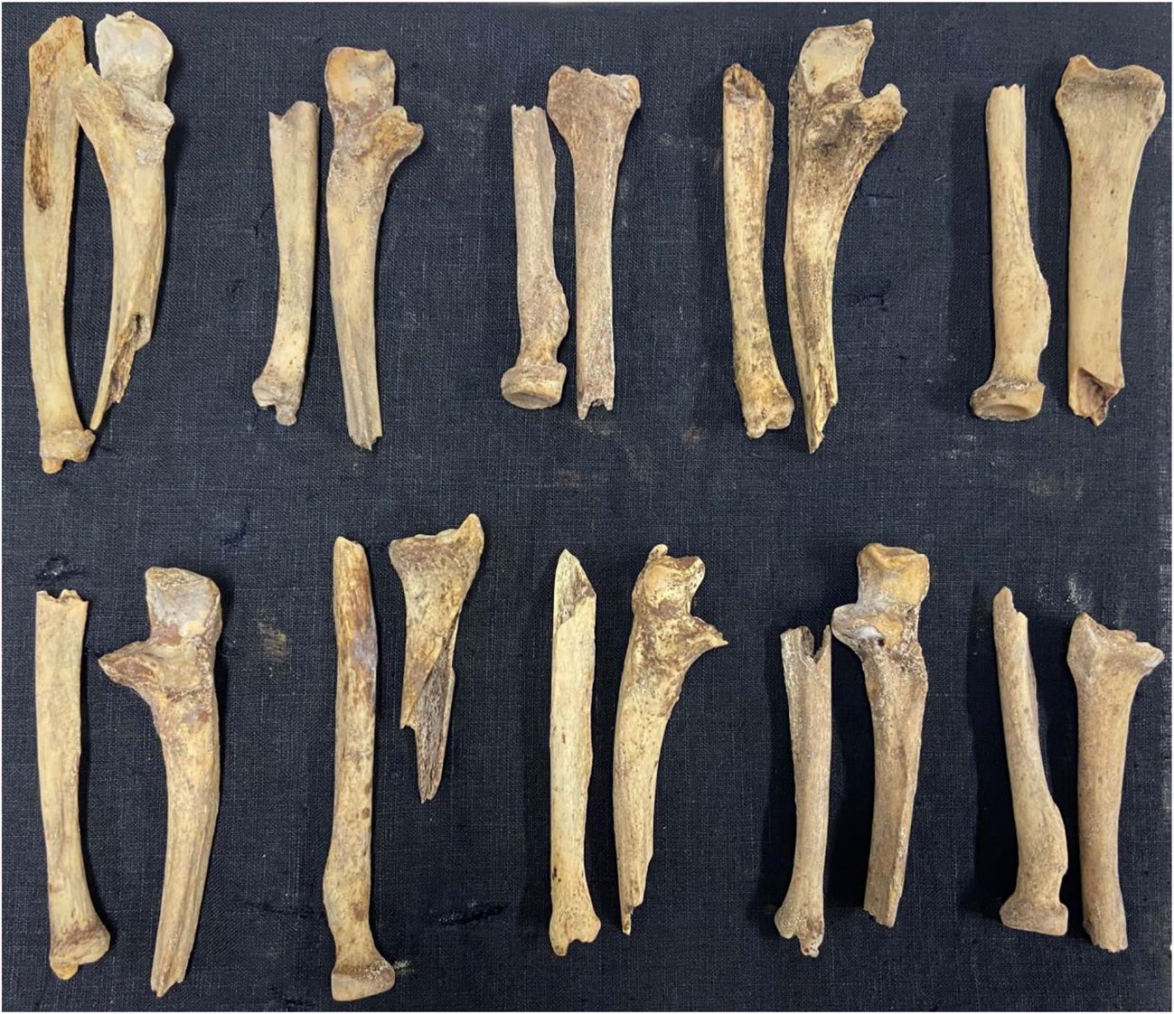
Six human ulnae and four human radii, broken into two bone segments. None were excluded for analysis

Six human ulnae and four human radii were used for this study. The bones were retrieved by manually defleshing human forearms, which were collected from body donors (see the section Ethics declaration) and kept at −20 °C prior to the study. After the defleshing process, the bones were macerated at 80 °C to remove all residual soft tissue and bone marrow. Each bone was then broken into two segments using a mechanical pendulum, resulting in a total of 20 unburned segments (for details regarding the pendulum see S. et al. (2022) [15]). A photograph of all bones is shown in Fig. 1. To establish the luminescence before burning, six of the 20 segments were randomly selected and photographed under white light and with the Foster + Freeman Crime-lite® 82S [16] 420-470 nm (blue, 445 nm peak) ALS excitation and the provided GG495 476 ± 6 nm (yellow) anti-glare Schott glass camera long pass filter. The images were not enhanced.

### Recreation of the spectral shift

To study the spectral shift, that is assumed to occur at above 800 °C, randomly selected bone segments from the collection, shown in Fig. 1, were burned at temperatures below and above 800 °C. Twelve of the 20 segments were grouped to be burned at 700 °C, and eight were grouped to be burned at 900 °C. A Carbolite Gero AAF 11/3 ashing furnace [17], available at the Glasinstrumentmakerij at Science Park, University of Amsterdam, was used. The bones were burned for 30 min and put on clean oven blocks to cool for another 30 min. Three segments were burned at once, in order to avoid excessive smoke production and prevent a temperature drop in the oven.

All bones, of both temperature groups, were analysed using the 420–470 nm (blue) Foster + Freeman Crime-lite® 2 handheld LED light source [18] and the 476 ± 6 nm (yellow) long pass filter for visualization of the luminescence, and compared in their periosteal surface luminescence. The findings were photographically documented using the setup and procedure described in section Visualizing luminescence and imaging and supplementary information S1. The raw images were processed using PhotoScape X 4.2.1 software for Mac devices, the background was darkened to black by manual selection, and converted to JPG format. No other changes, such as colour enhancements, were made to the images, and the white light images were not processed.

To test whether the spectral shift could be visualized on the same bone, two randomly chosen bone segments from the set burned at 700 °C for 30 min were burned for a second time at a higher temperature, of 900 °C, for an additional 30 min. The bones were analysed again using the 420–470 nm (blue) Foster + Freeman Crime-lite® 2 handheld LED light source [18] and 476 ± 6 nm (yellow) long pass filter, as well as photographed using the same equipment, setup and procedure as described in section Visualizing luminescence and imaging and supplementary information S1.

### Visualizing luminescence and imaging

The bones of each temperature group were laid out on a black textile sheet, a dark non-luminescent surface, which was laid over a foam panel, and analysed with the naked eye under white light. A Canon EOS 40D with an EF 100 mm f/2.8 Macro USM lens was used to take the images. Since the Foster + Freeman Crime-lite® 2 handheld LED light sources [18] provided only a small beam of light, the 420–470 nm (blue, peak at 445 nm) Foster + Freeman Crime-lite® 82S [16] was used for the photographs. The provided GG495 476 ± 6 nm (yellow) anti-glare Schott glass camera long pass filter was mounted onto the lens for the images taken with ALS excitation.

Additionally, direct comparison images of both groups were taken, containing two bones of each temperature group separated by a ruler, so that differences in the periosteal surface could be visualized more clearly and could be used later on for the colorimetric measurements. During the entire process, the samples were handled with care to prevent further fragmentation whilst wearing double nitril gloves to avoid contamination and potential injuries.

Elaboration on the imaging setup and used exposure times is found in supplementary information S1. The images were taken in raw image (CR) and JPG format.

### Colorimetric measurement and statistical analysis

Image J version 1.5326 was used to measure the percentage of red, green and blue colour in the images (.jpg) to quantify the change in colour after the spectral shift is seen to occur [19]. Eight bones of each temperature group (700 °C vs. 900 °C) were compared. Six measurements (ROIs: regions of interest) were generated for each bone, whereas each included the intensity of red, green and blue in the image. The contribution of each colour to the images was compared in-between bones burned at 700 °C and 900 °C and plotted using SPSS. The exact procedure and locations of the measured ROIs in each image are found in supplementary information S2. The group statistics (mean, standard deviation and error of each colour intensity) of the bones burned at 700 °C and 900 °C was calculated using SPSS. Additionally, an independent samples test was performed via SPSS. The intensities of red, green and blue in the group of bones burned at 700 °C and 900 °C were tested against each other. Significance was accepted at a *p* value of ≤ 0.05.

## Results

### Luminescence of periosteal surface—spectral shift

**Fig. 2:**
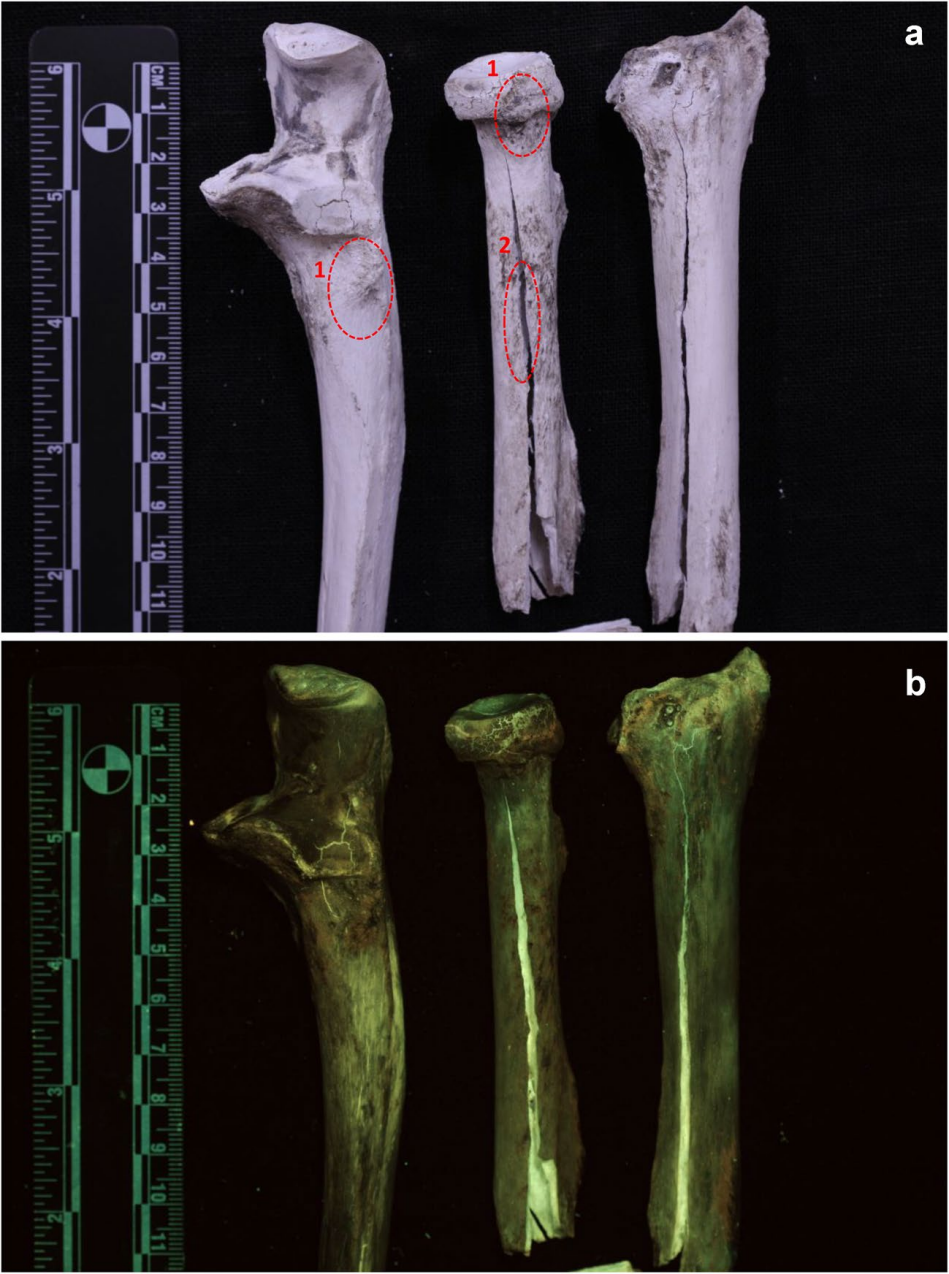
Periosteal surface of bone samples burned at 700 °C. A general green luminescence is observed. 1. grey/ashy areas – red/brown luminescence 2. longitudinal HIBFs – brighter green luminescence **a** samples under white light **b** samples excited with 420-470nm (blue; 476 nm long pass filter)

**Fig. 3:**
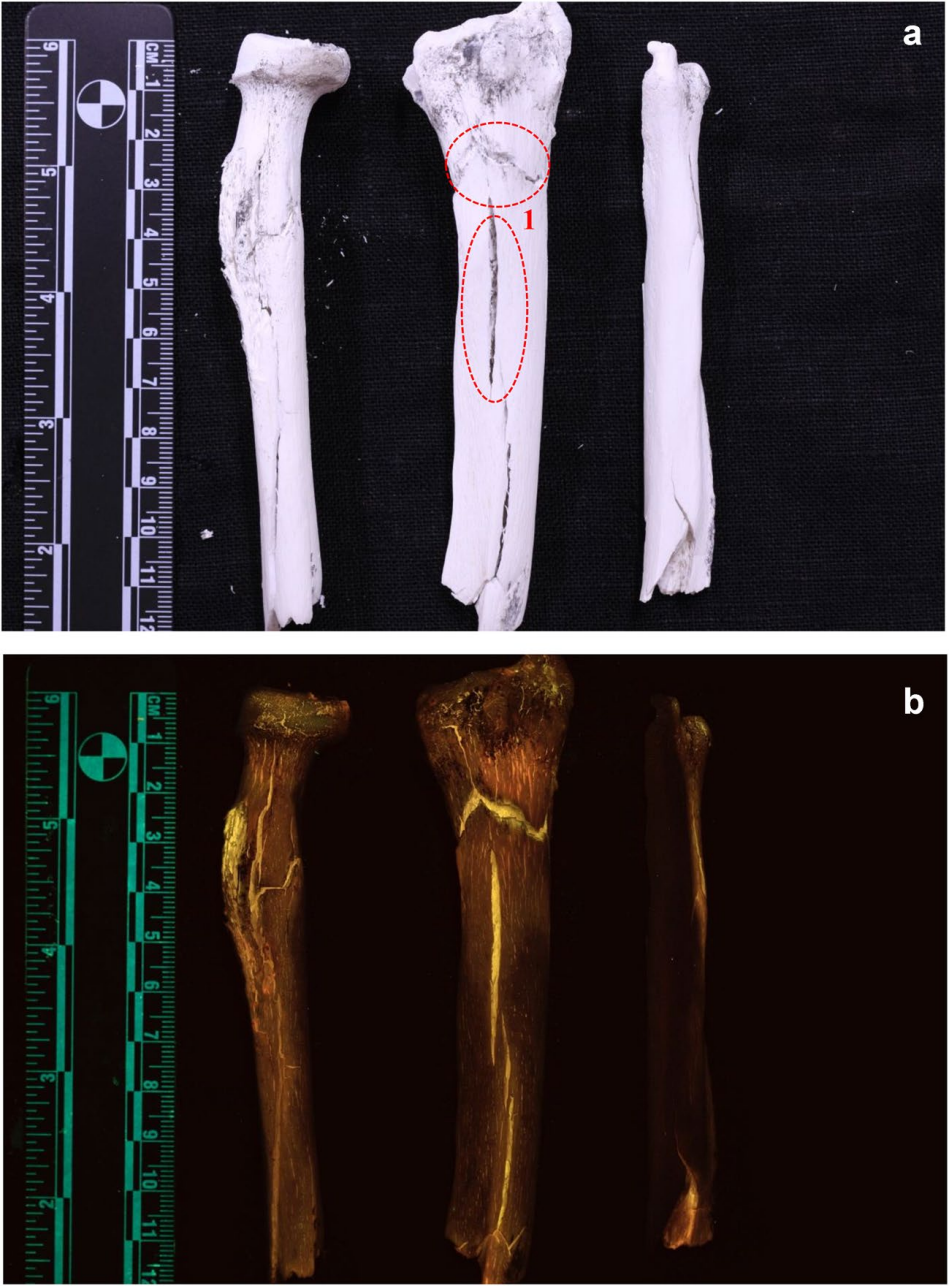
Periosteal surface of bone samples burned at 900 °C. A general red/brown luminescence is observed. 1. longitudinal and transverse HIBFs—brighter yellow luminescence **a** samples under white light. **b** samples excited with 420–470nm (blue; 476-nm long pass filter)

The unburned bone segments showed a uniform green luminescence in visually high observed intensity (see supplementary information, Fig. S3). All bone segments that were burned in the ashing furnace were calcined and white once the burning process was over. There was no difference in the stage of damage of the bone segments that were burned at 700 °C or 900 °C when analysed under white light (Figs. 2a and 3a). The bone segments were brittle and lighter in weight than before burning. When analysed with the 420–470 nm ALS, the segments burned at 700 °C showed an overall green luminescence on the periosteal surface (Fig. 2), although with significant decrease in visually observed intensity compared to the unburned bones. Heat-induced bone fractures (HIBFs) such as longitudinal or patina fractures on the periosteal surface showed higher luminescence intensity than the surrounding area (Figs. 2 and 3).

**Fig. 4:**
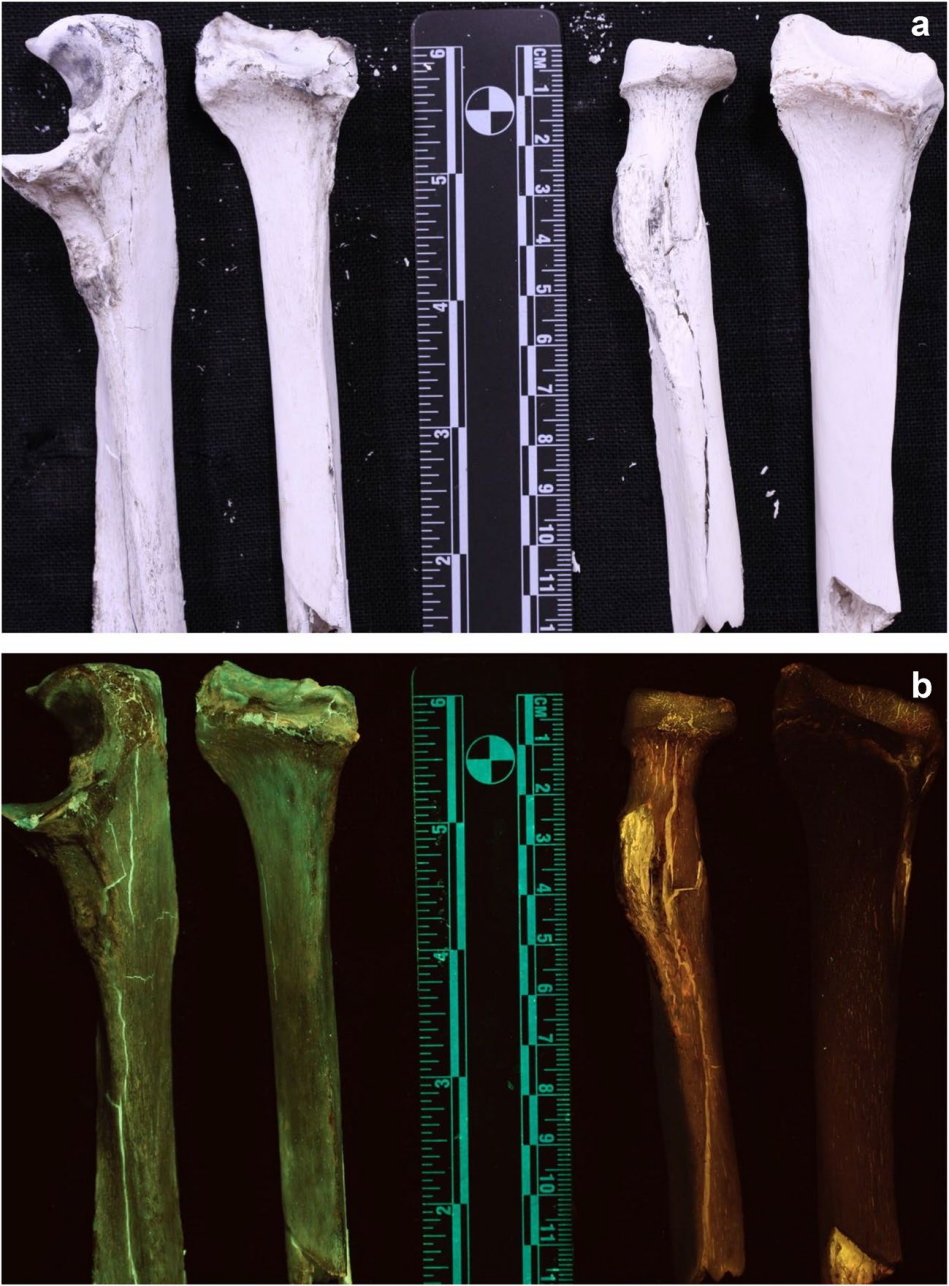
Direct comparison of periosteal surface of bones burned at 700 °C (left) and 900 °C (right). **a** samples under white light. **b** samples excited with 420—470nm (blue; 476-nm long pass filter)

The segments that were burned at 900 °C showed a different colour of luminescence when analysed with the 420–470 nm ALS. The periosteal surface showed red to brown luminescence with decreased visually observed intensity (Fig. 3). The colour of luminescence was more uniform than seen on the bones burned at 700 °C. The comparison images showed that there is a clear and distinct difference in the wavelength bandwidth of the emitted light between the bones burned at 700 °C and the ones burned at 900 °C (Fig. 4).

**Fig. 5:**
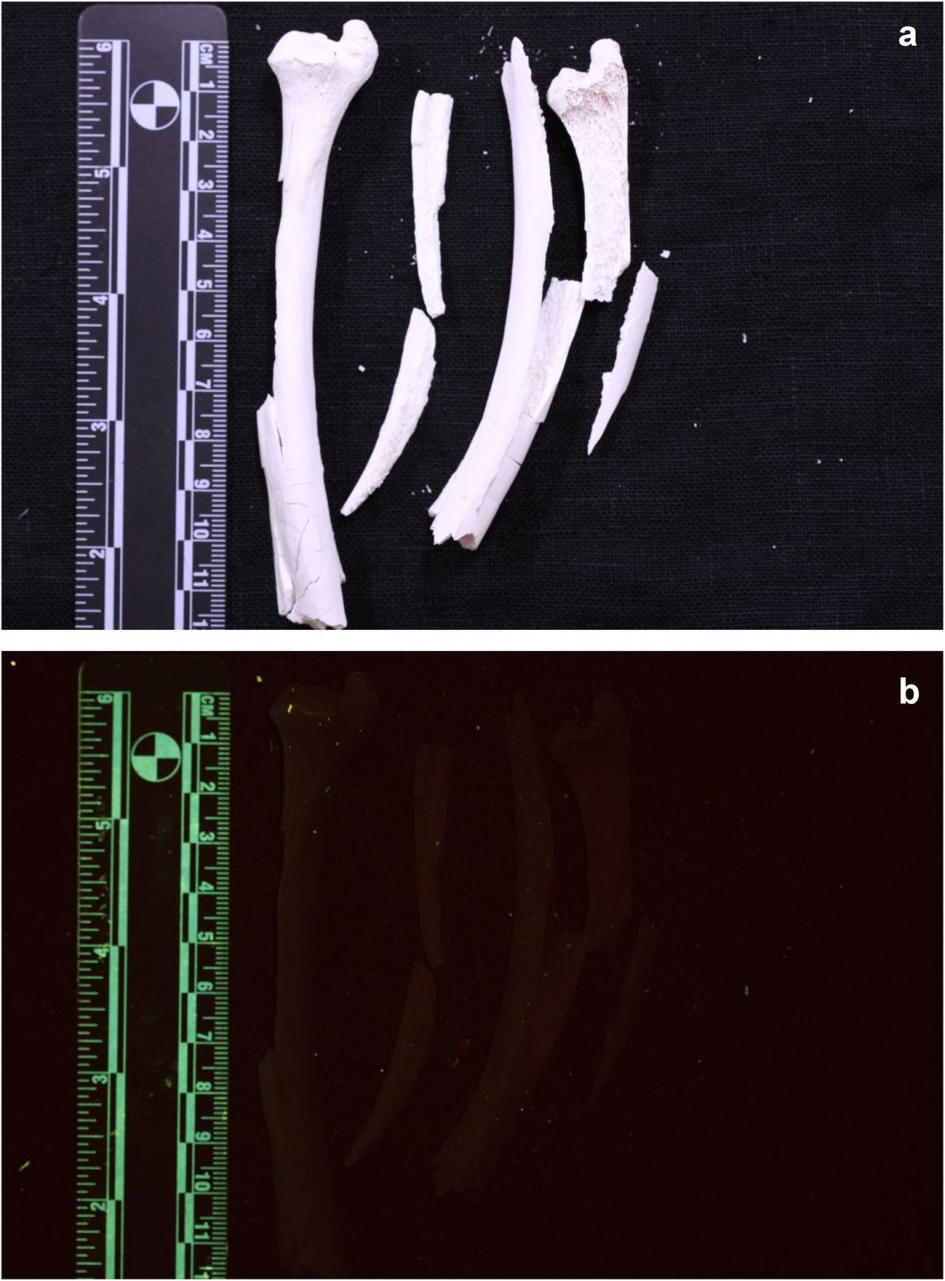
Bones previously burned at 700 °C and then again at 900 °C. After the second burning procedure, the bones no longer show a green luminescence. **a** samples under white light. **b** samples excited with 420-470 nm (blue; 476-nm long pass filter)

When analysing the two bone segments that were previously burned at 700 °C and then burned again at 900 °C, one can see that the bones that showed a green luminescence at 700 °C now showed a very low intensity of luminescence when burned at 900 °C (Fig. 5).

### Colorimetric measurement and statistical analysis

**Fig. 6:**
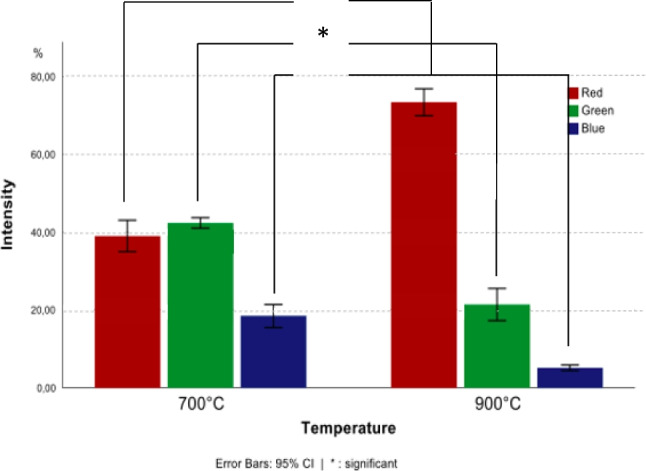
The plot of the colorimetric analysis shows significant changes of all three colour intensities between bones burned 700 °C and 900 °C

The percentages of the different colour intensities of the luminescence were plotted into a single graph, shown in Fig. 6. This plot shows that at 700 °C, green (495-570 nm) was represented in the images with the highest intensity at approx. 45%, followed by red (620-750 nm) and blue (450-495 nm). The intensity difference between green and red for bones burned at this temperature was not very large but still distinguishable. The plot of the bones burned at 900 °C shows that the largest contribution of the measured colours came from red, the percentage being just below 80%. There was a large difference when compared to the green intensity, as this only shows intensities slightly over 20%.

The Levene’s test for the equality of variances showed that the variances can be assumed to be equal. The *p* values of the *t* test are smaller than the significance level of 0.05, meaning the change of intensities in all three colours is significant (Table 1). The full output of SPSS can be found in Tables 1 and 2, in supplementary information S2.

**Table 1:** T test of equality of means of RGB intensities of bones burned at 700 °C and 900 °C. *p* values are below the significance level of 0.05, meaning the shift of colour intensities in the detected luminescence between these two temperature groups is significant

	*t*-test for equality of means							
	*t*	df	Significance		Mean difference	Std. error difference	95% CI of difference	
			*One-sided p*	*Two-sided p*			*Lower*	*Upper*
Red	−15.167	14	<.001	<.001	−34.35457	2.26506	−39.21263	−29.49651
Green	11.479	14	<.001	<.001	21.05409	1.83408	17.12038	24.98781
Blue	10.423	14	<.001	<.001	13.30048	1.27610	10.56353	16.03743

## Discussion

The intensity difference between unburned and burned bone was expected and seen in previous research; fire exposure reduces the observed luminescence intensity, primarily due to the loss of collagen, and chemical alterations to the inorganic bone matrix [9], [20]. The brittleness and weight loss are also consequences of these physical and chemical alterations of the bone[21], [22]. A possible explanation for the higher luminescence intensity in heat-induced cracks might be a shorter duration of heat exposure, resulting in less thermal alteration to fluorophores since fragmentation occurred during fire exposure. Fragmentation can occur at different times during the burning process, sometimes even after the cooling period through handling the bones [15].

The reproduced shift from green luminescence at 700 °C to red luminescence at 900 °C, in the experiments presented in this paper, confirms and substantiates the initial findings that there is indeed a spectral shift around the temperature threshold of 800 °C. This was further confirmed and quantified by colorimetric analysis. The largest contribution to the colour, and therefore observed luminescence, switched from green and red at 700 °C to most dominantly red at 900 °C. The colorimetric analysis using ImageJ provides an objective way in analysing and interpreting the observed difference in luminescence colour [19]. It is not certain what exactly causes this change in the emission wavelength bandwidth, but since the conditions and variables for the experiments were the same (i.e. same ashing furnace and exposure time of 30 min), it can be suggested that a large contributor to the change in luminescence was the variable that differed the most, the exposure temperature.

Fresh, unburned bone has a high luminescence intensity. Fire exposure to these bone samples causes an overall decrease in luminescence intensity [9]. Between 400 and 800 °C, the intensity of luminescence increases again, when compared to samples burned below 400 °C, excluding unburned samples [9]. This is said to occur due to the combustion of the remaining organic components in the bone matrix. At temperatures above 800 °C the intensity decreases again with increasing exposure times, possibly due to a change in the inorganic components of the bone matrix [9]. The result of the bone samples burned at 700 °C and again at 900 °C reflected this. One has to note that these bone samples were first exposed to 30 min at 700 °C heat, and additionally to 30 min at 900 °C. These samples were therefore exposed to a higher accumulated heat energy than the samples that were only burned once for 30 min, which most likely explains the diminished luminescence of these samples.

The change in emission wavelength bandwidth could be related to changes in the bone matrix. The most significant structural changes occur between 600 and 800 °C [23], [24] which could very well be a potential cause for the shift in luminescence. Since the collagen is already removed at these temperatures [25], a change has to occur in the bioapatite. The third and fourth stage of damage, inversion and fusion, set in at temperatures above 700°C; here, the inorganic component of the bone matrix is chemically altered as the carbonate substitutions with hydroxyl- and phosphate groups take place, and the apatite crystals grow and coalesce. At temperatures of about 800 °C, a specific alteration to the bone matrix must take place that induces this change in luminescence from the more ‘patchy’ and non-uniform green luminescence of the bones burned at 700 °C, to the almost entirely uniform red luminescence of bone segments burned at 900 °C. Results of X-ray diffraction (XRD) and Fourier transform infrared spectroscopy (FTIR) show that carbonate is no longer detected in calcined bones burned at 900 °C, whilst it is still present at 600 °C [25]. It seems that at 900 °C, the mineral crystallinity increases as the apatite crystals grow and the carbonate is completely removed from the apatite lattice [25]. XRD results have also shown that when heated above 800 °C, the crystals in the inorganic bone matrix change from their small and rod-like appearance to a more equidimensional and large crystal (increase in size by factor 3) [24]. Additionally, the CaO concentration increases with higher temperatures, although it is unknown whether CaO is the end product of oxidizing carbonate and detected at temperatures above 700 °C or 900 °C [23], [24]. This means that both the loss and substitution of carbonate with other molecules and the increase of crystal size could result in the change in luminescence colour and decrease of luminescence intensity seen at 900 °C. Further investigation using techniques such as the XRD or FTIR at temperatures between 700 and 900 °C, using smaller increments than 100 °C, could reveal further information on the changes to the inorganic apatite lattice.

The changes elaborated here are based on human bones being combusted under dioxygen rich conditions. The ashing furnace had an air inlet and chimney, which provided sufficient air flow to allow for combustion. Previous research has shown that the heat-induced changes experienced by bones burned in ashing furnaces and realistic cases (i.e. house fires) can be compared [9], [26]. The design of this experiment corresponds to construction and compartment fires, as a total lack of dioxygen in these cases is relatively uncommon. A temporary lack of dioxygen, however, can occur when the bones are in direct contact with the flame as the dioxygen levels are low within the flame. Since the flame front is dynamic, and moves over the materials, sections of bone will be exposed to radiating heat in an dioxygen rich environment when the flames have passed. However, the exposure of the bones to the heat is more uniform and standardized in the oven compared to the more dynamic and inhomogeneous environment of, for example, a house fire. The results of this experiment, however, cannot be completely compared to a deoxygenated environment. Previous research has shown that when bone is exposed to heat in the absence of dioxygen, the physical and chemical heat-induced changes are different [7], [27], [28]. As a result, the observable and measurable colour change from ivory to black, brown and white under an atmospheric dioxygen level halts at carbonization in a deoxygenated environment. Due to the formation of amorphous carbon, without the possibility of reacting with dioxygen to form carbon dioxide or carbon monoxide, the bone remains brown/black even in high temperatures up to 1000 °C [7], [29]. This means that the luminescence of bone is not observed for these samples, as the carbon absorbs the excitation and possibly the emission light [9]. Therefore, the results of this study only apply to bone burned under oxidizing conditions that have reached the calcination stage.

Practically, the different colours of luminescence and statistical differences in the colour intensities at different temperatures observed in this research could aid in inferring information on the fire temperature that the bones were exposed to. It is, however, challenging to replicate exact case scenarios, meaning the results and their interpretations have to be applied to forensic cases with caution. Different exposure times, burning temperatures and patterns, as well as the environment of the fire site can alter the state of damage to the bones, and therefore the possible observed luminescence. There are variations in bone that have to be considered, such as differences in the weight or bone mineral density (BMD), as well as age, sex, lifestyle and disease of the donor that can cause differences in burning behaviour, observed luminescence and fracture propagation. More research is needed to identify the exact influences of variable factors of the bone, of the fire and of the perimortem events on the detected luminescence to be able to explain its occurrence and origin. Additionally, ALS can be used for visualization and analysis of another heat-induced change, which is the occurrence of a heat border line (HBL) [30]. Receding soft tissue, or fire debris, can shield parts of the bone whilst the other is exposed to the flame. This creates burning patters such as the HBL, separating unburned from burned bone [2]. Although this is a predictable pattern, it can be influenced by factors such as body position, soft tissue and oxygen availability. This pattern disappears to the naked eye once the entire bone has reached the last stage of heat-induced damage and is calcined and therefore white. The calcination of the bone also leads to challenges in estimating exposure temperatures based solely on the colour of the bone. It is assumed that differences in exposure temperature or exposure duration of these calcined bones can still lead to burning patterns. The authors of this paper describe this as a latent HBL; a burning pattern that becomes invisible to the naked eye. The authors believe that the luminescence of burned bones and ALS can be used for visualization and analysis of latent HBLs, as the differences in fire exposure, temperature combined with exposure duration, can result in differences in luminescence on the same bone segment.

## Conclusion

This research provided substantiation for the hypothesis that burned human bones undergo a spectral shift of dominantly green to dominantly red luminescence at the threshold of around 800 °C. The shift from green to red due to a 200 °C difference in exposure temperature was documented in multiple samples and quantified with objective statistical analyses showing a significant difference in colour intensities at 700 °C and 900 °C, creating two colour intensity clusters. Using ALS to analyse burned human remains found on a fire scene allows for the visualization of relevant information that would otherwise be lost to the naked eye. The temperature specific green and red colour of luminescence of calcined bones progresses the understanding of fire-induced changes of human bone, thereby also aiding in the estimation of exposure temperature and the reconstruction of fire events. The findings of this research can help improve the work of a forensic anthropologist and add another objective technique to the toolbox that can be used in the investigations of human remains. By extracting more information from salvaged bones, complex environments, like fire incidents, can be deciphered and reconstructed more accurately. This will improve the workflow of forensic and criminal procedures and help in the investigations and work of the Criminal Justice System.

## Supplementary information

ESM 1 (DOCX 3583 kb)

## Declarations

Human bone samples were used for this research, obtained through the Amsterdam Universitair Medische Centra’s (AUMC) body donation program (BDP) of the Department of Anatomy, Embryology and Physiology of the Academic Medical Centre of Amsterdam, the Netherlands, in accordance with the Dutch Burial and Cremation Act (art. 67 Burial Act) and with approval of the ethical committee of the department of Medical Biology.
