# Supplementary material for: Temperature-specific spectral shift of luminescing thermally altered human remains
Source: Int J Legal Med. 2023 May 13;137(4):1277–86. doi: 10.1007/s00414-023-03006-0 (PMC10247558; doi:10.1007/s00414-023-03006-0)
Supplement: Supplementary file 1 — (DOCX 3583 kb) [file 414_2023_3006_MOESM1_ESM.docx]

# **Supplementary Information**

**S1 Elaboration on Photography Process of Analysed Bone Fragments**

The camera was mounted on a stationary camera stand with adjustable height at 102.8 cm. The Crime-lite^®^ 82S was mounted on a laboratory stand, held at 55 cm height with a double socket and stand clamp. The light was held parallel over the bones on the foam panel, making sure all fragments were equally illuminated (Fig. S1). The bones were put in position on the black sheet, white light images were taken first, then the filter was mounted on to the camera and the ALS images were taken next. The process was repeated for two sides of the bone, resulting in two white light and two ALS images for each bone. It is worth to note that previous research has shown that the actual output bandwidth of the used ALS exceeds the stated bandwidth


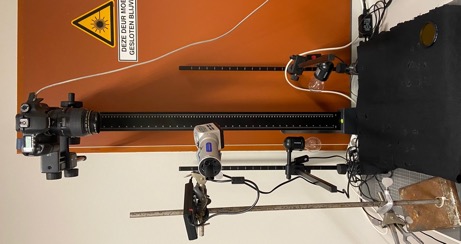
All white light images were taken with 0.4 s exposure time. The ALS images had to be taken with different exposure times, as the intensity of observed luminescence was different in each case. The ALS images of the unburned bones were taken with 1/5 s exposure time, as the intensity was fairly high. The ALS images of the bones burned at 700° were taken with 1 s exposure time, whereas the bones burned at 900°C were taken with 1.3 s.

**Fig. S1**: Camera setup used for all photographs.

Lens height: 102.8 cm

ALS height: 55 cm

## **S2 Colour Measurement Procedure using Image J and Statistical Analysis**

The colorimetric analysis was performed on the comparison images taken of the 700°C and 900°C bones. As the white balance was set on automatic, the temperature and colour of the used images was manually changed to the same value in Lightroom to ensure equal intensity conditions throughout all images.

A total of eight bones per temperature, resulting in eight images were analysed. The images were loaded onto Image J, in which they were first made into RGB composites. The red, green and blue colour intensity in three 180 x 180 ROIs per bone per side were measured, resulting in six measurements per bone. Each colour intensity of each ROI was turned into a percentage which were then averaged for a single bone. The intensities for the respective colours were then averaged across all bones for each temperature and plotted in a single graph using SPSS. An independent samples test was performed with SPSS to test whether the change in colour intensity was significant (Table 1 and 2). The following images show where the ROIs were selected. Areas with intense fragmentation were avoided when possible as these deviated in luminescence compared to the periosteal surface.

Table 1: Overview of the mean, standard deviation and error of the RGB intensities of eight bones burned at 700°C and 900°C.

| **Group Statistics** | | | | | |
| --- | --- | --- | --- | --- | --- |
|  | Group | N | Mean | Std. Deviation | Std. Error Mean |
| Red | 700 | 8 | 39,0698 | 4,88099 | 1,72569 |
|  | 900 | 8 | 73,4243 | 4,14967 | 1,46713 |
| Green | 700 | 8 | 42,4551 | 1,61746 | ,57186 |
|  | 900 | 8 | 21,4010 | 4,92896 | 1,74265 |
| Blue | 700 | 8 | 18,4752 | 3,50545 | 1,23937 |
|  | 900 | 8 | 5,1747 | ,85974 | ,30396 |

| **Independent Samples Test** | | | | | | | |
| --- | --- | --- | --- | --- | --- | --- | --- |
|  | | t-test for Equality of Means | | | | | |
|  |  | Significance | | Mean Difference | Std. Error Difference | 95% CI of Difference | |
|  |  | One-Sided p | Two-Sided p |  |  | Lower | Upper |
| Red | Equal variances assumed | <,001 | <,001 | -34,35457 | 2,26506 | -39,21263 | -29,49651 |
|  | Equal variances not assumed | <,001 | <,001 | -34,35457 | 2,26506 | -39,22446 | -29,48468 |
| Green | Equal variances assumed | <,001 | <,001 | 21,05409 | 1,83408 | 17,12038 | 24,98781 |
|  | Equal variances not assumed | <,001 | <,001 | 21,05409 | 1,83408 | 16,86683 | 25,24135 |
| Blue | Equal variances assumed | <,001 | <,001 | 13,30048 | 1,27610 | 10,56353 | 16,03743 |
|  | Equal variances not assumed | <,001 | <,001 | 13,30048 | 1,27610 | 10,34725 | 16,25371 |

| **Independent Samples Test** | | | | | |
| --- | --- | --- | --- | --- | --- |
|  | | Levene's Test for Equality of Variances | | t-test for Equality of Means | |
|  |  | F | Sig. | t | df |
|  |  |  |  |  |  |
| Red | Equal variances assumed | ,839 | ,375 | -15,167 | 14 |
|  | Equal variances not assumed |  |  | -15,167 | 13,647 |
| Green | Equal variances assumed | 3,142 | ,098 | 11,479 | 14 |
|  | Equal variances not assumed |  |  | 11,479 | 8,490 |
| Blue | Equal variances assumed | 7,381 | ,017 | 10,423 | 14 |
|  | Equal variances not assumed |  |  | 10,423 | 7,839 |

Table 2: Output of the independent samples test using SPSS. The Levene’s Test for Equality of Variances shows that the variances can be assumed to be equal. The t-test for equality of Means shows that the change of intensities in RGB from the bones burned at 700°C to 900°C are significant.


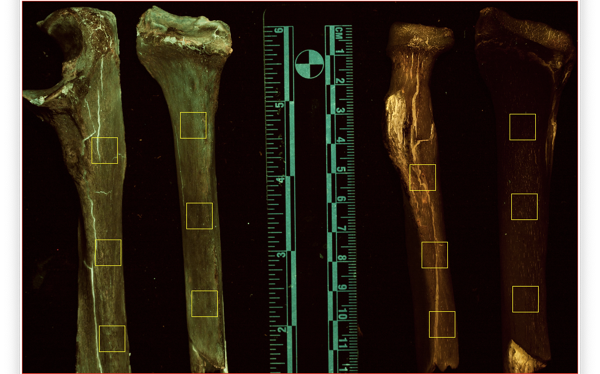

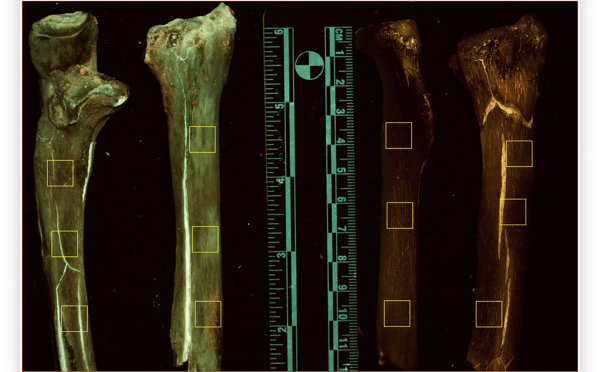


A

B

##
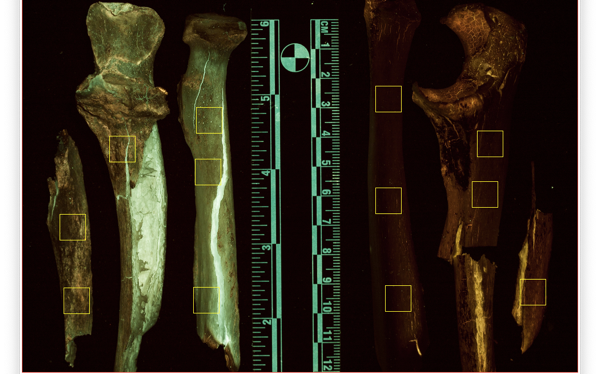


C


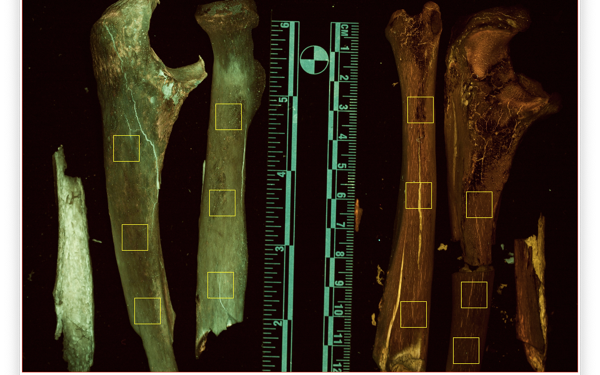


D


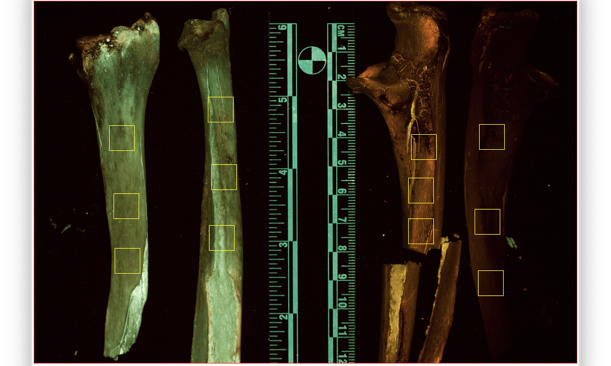

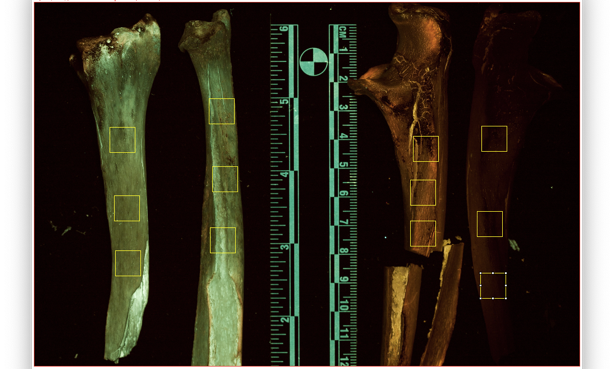


E

F


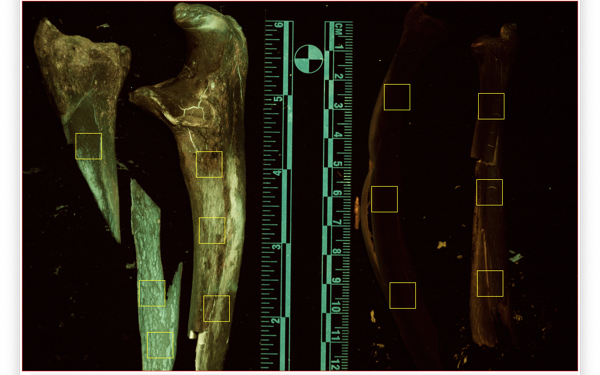

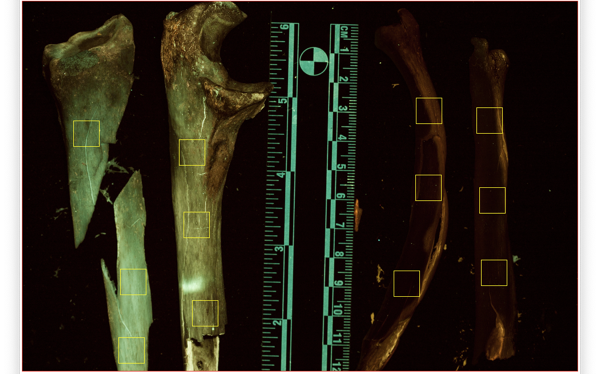


G

**Figure S2 A-H:** Selected ROIs (180x180) for colorimetric analysis using Image J on images taken with ALS excitation.

H

## **S3 Luminescence of Unburned Bones**


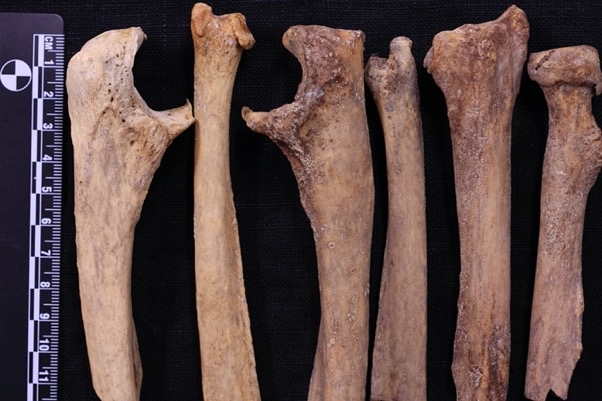
The following images show photographs taken from the analysed bones before they were burned. A random selection of fragments was photographed, front and back, under white light and with 420-470 nm blue light and 476 nm long pass filter. All bones showed a general green luminescence.

A


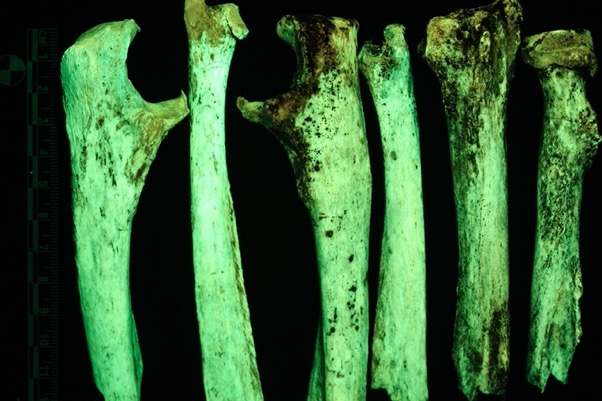


B


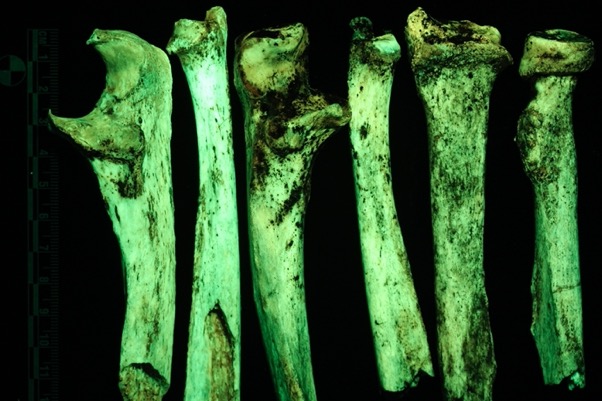

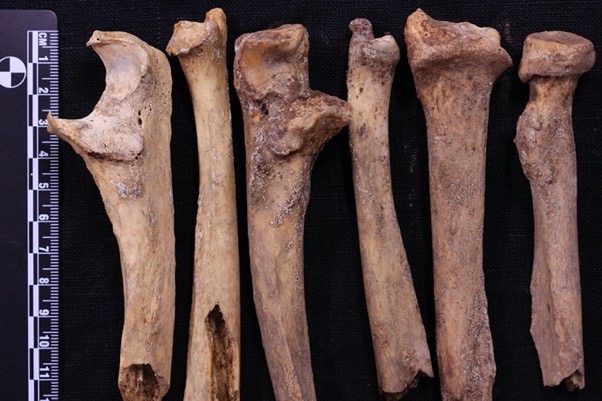


**Figure S3:** Periosteal surface of unburned bone segments.

A: Front, white light B: Front, 420-470 nm (476 long pass filter)

C: Back, white light D: Back, 420-470 nm (476 long pass filter)

C

D
